# COVID-19 preventive behaviors and influencing factors in the Iranian population; a web-based survey

**DOI:** 10.1186/s12889-021-10201-4

**Published:** 2021-01-15

**Authors:** Mojgan Firouzbakht, Shabnam Omidvar, Saeedeh Firouzbakht, Arman Asadi-Amoli

**Affiliations:** 1 Department of nursing- midwifery, Comprehensive Health Research Center, Islamic Azad University Babol Branch, Babol, Iran; 2 Social Determinants of Health Research Center, Health Research Institute, Babol University of Medical Sciences, Babol, Iran; 3 Bushehr University of Medical Sciences, Bushehr, Iran; 4 Student of research committee, Babol University of Medical Sciences, Babol, Iran

**Keywords:** Preventive behaviors, Online survey, COVID-19, Web-based

## Abstract

**Background:**

COVID19 is a respiratory disease caused by a novel coronavirus. As there has been no definitive treatment for the disease so far, the only way to control the spread is to break the chain of infection. Our study aimed to analyze the preventive behaviors and influencing factors in the Iranian population.

**Methods:**

This cross-sectional study was a web-based survey in the Iranian population. We performed the study during the first peak of COVID-19 outbreak (from March 25th, 2020 to April 5th^)^. We used demographic and Preventive behaviors questionnaires to collect the data. This web-based survey was publicized on the internet through the common platforms used by the Iranian population. This survey was released on the website “Porsline.com”. A total of 2097 acceptable questionnaires were filled. All data were analyzed, using Statistical Package for Social Sciences (SPSS) version 19.

**Results:**

61.9% of the participants checked the hand-washing question as “Always”. 55.7 and 58.2% checked the wearing masks and gloves as “Always”, respectively. We found a significant relationship between gender and hand washing behavior (*P* = 0.006) and the use of masks and gloves (*P* < 0.001). Results showed that wearing gloves had a significant relation with the education status (*P* = 0.029) and economic status (*P* = 0.011). Wearing masks had a significant relation with economic status (*P* = 0.032). Overall women had better preventive behaviors.

**Conclusions:**

Preventive behaviors have a significant relation with some socio-demographic characteristics. According to the 3 main preventive behaviors of hand-washing, wearing masks and gloves 50% of the population has not taken these behaviors seriously.

**Supplementary Information:**

The online version contains supplementary material available at 10.1186/s12889-021-10201-4.

## Background

COVID19 is a respiratory disease caused by a new coronavirus, first found in December 2019 in Wuhan, China. The disease is highly contagious, and its symptoms include fever, fatigue, dry cough, muscular pain, and dyspnea. It has been reported that 18.5% of the Chinese patients, developed to the severe stage, which leads to Acute Respiratory Distress Syndrome (ARDS), Acidosis, Septic Shock, Bleeding, and Disseminated Intravascular Coagulation (DIC) [1, 2].

Different medications were used in various treatment protocols to experiment with lethality control of the disease in clinical trials. Some of these medications proved to reduce lethal complications [3, 4].

The epidemic outbreak of COVID19 was so fast that it had spread to 26 different countries by February 15, 2020 [5] and by the end of the same month, 85,681 cases and 2933 deaths were reported; most of which were reported by China [6]. On January 30, 2020, the World Health Organization (WHO), declared this epidemic outbreak as a Global Health Emergency [7]. On 8th of April 1,282,931 confirmed cases, 72,616 confirmed deaths were reported by WHO [8]. The first diagnosed cases in Iran were confirmed on February 19, 2020 in Qom, and the rapid spread of the disease caused the contagion of more states. The states of Golestan, Mazandaran, Tehran, and Isfahan were reported as red regions [9].

COVID-19 is transmitted from person to person through direct contact, infected surfaces, droplets, and fecal-oral contact, and the incubation period ranges from 2 to 14 days. According to a report by WHO, by April 14, 2020, over 1 million cases and 50,000 deaths had occurred, since the outbreak of this disease; which is fearsome statistics.

Health is one of the basic rights of human beings and is generally considered a personal duty. The Self-care ability of each person is highly affected by factors, such as age, lifestyle, health status, emotional status, and knowledge. Self-care is defined as activities and precautions which a person takes, to prevent or control infections; in other words, precautions that are taken without professional help [10, 11]. In most the infectious disease, self-hygiene is the cheapest and easiest way to prevent infections.

By the end of January 2020, WHO and the Centers for Disease Control and Prevention (CDC), published a series of recommendations to prevent the spread of COVID19 [12, 13].

As there has been no definitive treatment, reported for this disease, the only way to control the spread is to break the chain of infection. Everyone has to stay home and follow the self-care guidelines that were recommended by the WHO; however, there is no data relating the preventive behaviors of the Iranian population, Hence, this study aimed to analyze the preventive behaviors of the Iranian population.

## Methods

### Study design

This cross-sectional study was conducted among the Iranian population. To prevent the spread of COVID-19 through contact, the data we collected through a web-based survey. This web-based survey was broadcasted on the internet through the common platforms used by the Iranian population. This survey was released on the website “Porsline.com“https://survey.porsline.ir/#/survey/56871/build. Inclusion Criteria included self-reported Iranian nationality and age 16 years and above.

### Data collection

The participant answered the questions anonymously, from March 25th, 2020 to April 5th. All study participants answered the socio-demographics and preventive behavior questions. This web-based questionnaire was filled, completely voluntarily. To ensure the quality of the survey, we set the 5-point-Likert (Always, most of the time, sometimes, rarely, never). The questionnaire that was filled in less than 1 min or more than 15 min, were excluded. The questionnaire was observed by 3500 and a total of 2097 questionnaires were filled.

## Measures

### Demographic information

Demographic variables including the name of the state, gender (male, female), age (years), marital status (single, married, widowed, divorced), education (High school, Graduate, Postgraduate.), occupation (Unemployed, worker/farmer, Government employee, Self-employed, student), economic status (Good, Average, Bad) were asked in this section.

### Preventive behaviors

The questionnaire was designed by the authors, with attention to preventive guidelines, presented by the Iranian ministry of health and treatment and WHO [12]. The questionnaire had 33 questions in four domains. Individual behavior (13 questions), guidelines for entering and leaving the home (8 and 8 questions, respectively), preventive guidelines for using personal belongings (4 questions). As COVID19 is a rapidly spreading and highly contagious disease, the participants that answered “always” are given “1”, and the other choices are scored as “0”; hence, the scores range from 0 to 33; where higher score indicated better preventive behavior. We determined the validity and reliability of the questionnaire. Eleven experts rated the items, and the content validity indices (CVI) of the questionnaire were calculated to be 0.81. The Cronbach’s alpha of the questionnaire was 0.82, indicating internal consistency.

### Statistical analysis

To identify factors influencing COVID-19 preventive behavior, multiple linear regression analysis was performed, and to determine the odds of preventive behavior, Logistic regression analysis was performed. All data were analyzed, using Statistical Package for Social Sciences (SPSS) version 19. *P*-values of less than 0.05 were considered statistically significant (2-sided tests).

## Results

This cross-sectional study was conducted in a way that 31 states contributed to the data for the study. The states of Tehran (24.9%) and Mazandaran (22%) had the highest contribution to the data pool; contrary to the state of Ilam (0.1%) with the lowest contribution. According to Porsline online survey system, the responding rate was 78% and the average duration of filling the survey was 5 min and 13 s. The questionnaire was filled by 2097 people, where 73% of them were directed to the survey through the online messaging app (98% of the surveys were filled by mobile phones). The participants’ general characteristics and COVID- 19 related preventive behavior were analyzed using frequencies, percentages, mean, and standard deviations. Differences in preventive behavior were analyzed according to general characteristics, utilizing independent t-test, ANOVA, and post-hoc Scheffe test.

The mean age of the participants was 34.85 ± 10.26 years, and 71.6% of them were women. Preventive behaviors were studied in an overall analysis, as well as in 4 separate domains. The mean overall score was 22.46 ± 6.38 out of 33. The relation between the preventive behavior domains and socio-demographic characteristics is presented in Table 1.

**Table 1:** Comparison of demographic characteristics by preventive behavior domains

			Preventive behavior											
			Individual behavior			Entering guidelines			Leaving guidelines			Personal belongings’ guidelines		
		N (%)	Mean (SD)	*T/F	P	Mean (SD)	*T/F	P	Mean (SD)	*T/F	P	Mean (SD)	*T/F	P
Gender	Female	1503 (71.7)	8.12 (2.40)	9.84	<.001	6.038 (5.205)	9.16	<.001	5.423 (1.891)	13.06	<.001	3.859 (1.41)	8.65	<.001
	Male	594 (28.3)	6.93 (2.70)			5.205 (2.107)			4.161 (2.133)			3.237 (1.65)		
Age	< 20	83 (4)	7.59 (2.55)	1.56	.166	5.54 (1.93)	4.72	<.001	5.03(2.18)	3.46	.004	3.61 (1.56)	1.68	.135
	20–30	698 (33.8)	7.64 (2.61)			5.59 (1.98)			4.81 (2.08)			3.66 (1.46)		
	31–40	775 (37.5)	7.95 (2.44)			5.85 (1.88)			5.15 (2.02)			3.76 (1.49)		
	41–50	330 (16)	7.81 (2.56)			6.17 (1.72)			5.31 (1.97)			3.65 (1.55)		
	51–60	152 (7.4)	7.72 (2.65)			5.75 (1.98)			5.14 (1.94)			3.55 (1.58)		
	> 60	30 (1.5)	7.23 (2.67)			5.6 (2.11)			5.33 (2)			3.06 (1.68)		
Marital status	Single	775 (37)	7.72 (2.57)	.70	.54	5.57 (1.98)	5.91	.001	4.83 (2.08)	5.51	.001	3.60 (1.49)	1.91	.12
	Married	1242 (59.2)	7.81 (2.55)			5.92 (1.87)			5.19 (2.01)			3.72 (1.52)		
	Divorce	64 (3.1)	8.5 (1.89)			6.31 (1.53)			5.43 (1.75)			3.31 (1.49)		
	Widowed	16 (.8)	7.93 (2.42)			5.96 (1.60)			5.32 (1.79)			3.93 (1.40)		
Education	High school	449 (21.3)	7.47 (2.65)	4.43	.012	5.71 (1.97)	.814	.443	5.10 (2.03)	2.18	.113	3.52 (1.59)	5.52	.004
	Graduate	1036 (49.4)	7.88 (2.58)			5.85 (1.89)			5.13 (2.03)			3.78 (1.47)		
	Postgraduate	615 (29.3)	7.85 (2.39)			5.78 (1.89)			4.92 (2.05)			3.62 (1.49)		
Occupation	Unemployed	613 (29.2)	7.89 (2.52)	3.30	.010	5.94 (1.78)	2.33	.054	5.35 (1.89)	5.18	<.001	3.85 (1.49)	4.32	.002
	Farmer/worker	37 (1.8)	6.78 (2.46)			5.21 (1.88)			4.41 (2.14)			3.29 (1.57)		
	Government Employee	669 (31.9)	7.87 (2.58)			5.81 (1.95)			5.00 (2.11)			3.59 (1.530		
	Self employed	451 (21.5)	7.52 (2.62)			5.72 (1.97)			4.87 (2.07)			3.54 (1.54)		
	student	327 (15.6)	7.91 (2.40)			5.67 (1.94)			4.99 (2.04)			3.75 (1.38)		
Economic Status	Good	400 (19.1)	8.17 (2.41)	10.14	<.001	6.07 (1.75)	9.9	<.001	5.38 (1.90)	5.79	.003	3.77 (1.48)	.835	.434
	Average	1431 (68.2)	7.77 (2.52)			5.80 (1.90)			4.99 (2.04)			3.65 (1.51)		
	Bad	266 (12.7)	7.27 (2.78)			5.40 (2.08)			4.97 (2.16)			3.68 (1.53)		
Chronic health condition	Yes	175 (8.3)	7.78 (2.62)	−.19	.84	5.81 (2.06)	.18	.85	5.04 (2.02)	−.29	.76	3.71 (1.51)	−.16	.87
	No	1504 (71.7)	7.82 (2.50)			5.78 (1.87)			5.09 (2.01)			3.73 (1.47)		

*Independent t-test/ANOVA

Among the individual preventive behaviors, the most emphasized ones (Washing the hands for at least 20 s, wearing a mask, and wearing gloves) were studied separately. 61.9% of the participants checked the hand-washing question as “Always”, however, 51.7 and 41.8% of them, did not check wearing gloves and masks as “Always”, respectively (Table 2). We found a significant relationship between gender and hand washing behavior (*P* = 0.006) and the use of masks and gloves (*P* < 0.001). Women had better preventive behavior in all domains. We also found that wearing gloves had a significant relation with the education status (*P* = 0.029) and economic status (*P* = 0.011). Wearing masks had a significant relation with economic status (*P* = 0.032).

**Table 2:** Percentage of positive response to preventive behavior

Items	Positive response N(%)
**Individual behaviors**	
I do NOT leave home, unless it is necessary	935 (44.6)
I avoid handshakes and hugging others	1845 (88)
I keep a minimum distance of 1.5 m from others	980 (46.5)
I avoid touching my face (eyes, nose and mouth)	345 (31.2)
I regularly wash my hands for AT LEAST 20 s	1298 (61.9)
I wear disposable gloves when I leave the house	1013 (48.3)
I cover my mouth and nose while sneezing or coughing	1572 (75.2)
I dispose of tissue papers in a lidded trash can	1461 (69.7)
I DO NOT visit friends and relatives	1268 (60.5)
I DO NOT attend birthday parties, wedding parties, or any other parties	1657 (79)
I DO NOT eat out	1764 (84.1)
I DO NOT use public transportation	1885 (89.9)
I disinfect my work space surfaces before anything else	1546 (73.7)
**Practices when entering the house**	
I wash my hands before taking off my clothes or do any other task	1571 (74.9)
I dry my hands using tissue papers	1967 (93.8)
I dispose of the tissue paper in a lidded trash can	1112 (53)
I disinfect my belongings such as cellphone, keys, wallet, etc. using alcohol disinfectant (70% alcohol)	1461 (69.7)
I hang my clothes separately from other clothes when I enter the house	1513 (72.2)
I wash my hands again, after removing my clothes	1281 (61.1)
I wash my hands after using the WC and before eating	1380 (65.8)
I disinfect all surfaces, everyday	1883 (89.8)
**Practices when leaving the house**	
I ask myself about the necessity, when I am leaving the house	926 (44.2)
I take alcohol disinfectant with me	1298 (61.9)
I wear disposable masks	1169 (55.7)
I wear disposable gloves	1221 (58.2)
I do not leave the house in case I have symptoms of fever and cough	1249 (59.6)
I leave the house wearing a mask, in case I have symptoms of fever and cough	1695 (80.8)
I always carry clean tissue papers	1501 (71.6)
I do not touch elevator buttons with bare hands	1414 (67.4)
**Practices while using personal belongings**	
I do not take my cell phone out of my pocket when outside, unless there is an emergency	1243 (59.3)
I do not remove my glasses or wrist-watch when outside	1464 (69.8)
I do not place my belongings on surfaces I am not certain of their hygiene	1615 (77)
I take food (Meat, chicken, eggs, etc.) only in a well done doneness	1856 (88.5)

Linear regression analysis was used to study the predictive behaviors. In linear univariate regression analysis, age (*P* = 0.016), Gender (*P* ≤ 0.001), economic status (*P* = 0.001) were the predictive variables, and in multivariate linear regression analysis, gender, economical status (P ≤ 0.001) and age (P = 0.001) were the predictive variables. Table 3 shows that the adjusted linear regression variables, predict the domains.

**Table 3:** Adjusted model linear regression predictive prevention behavior against covid-19 in four domains. (*n* = 2097)

variables	Individual behavior			Entrance behavior			Leaving protocols			Personal belongings		
	β	t	*p*	β	t	*p*	β	t	*p*	β	t	*p*
Constant		22.93	<.001		26.61	<.001		27.835	.000		28.581	<.001
Gender	−.199	−8.242	<.001	−.187	−7.741	<.001	−.279	−11.61	.000	−.166	−6.824	<.001
Economic status	−.087	−3.56	<.001	−.097	−4.049	<.001	.114	4.733	.000			
Education	.056	2.287	.022			<.001						
Age	.054	2.225	.026	.101	4.173	<.001						
Marital status										.048	1.96	.049

Logistic regression analysis was performed to assess odds ratio of preventive behaviors according to socio-demographic in Table 4. We found that the chance of appropriate preventive behaviors in all the domains was two times better in women than men. Other variables are presented in the table.

**Table 4:** Adjusted OR (CI 95%) variables effected prevention behavior against covid-19 (*n* = 2097)

		Individual behavior			Entering Protocols			leaving protocols			Personal belongings protocols		
		OR	CI 95%	*p*	OR	CI 95%	*p*	OR	CI 95%	p	OR	CI 95%	*p*
Gender	Female vs. male	1.67	1.288–2.168	<.001	1.99	1.57–2.51	<.001	2.67	2.005–3.567	<.001	1.804	1.44–2.25	<.001
Education	<Graduate vs. >Postgraduate	.726	.566–.931	.012	.798	.639–.998	.048						
Economic status	Good vs. Bad	1.47	1.11–1.93	.006									
Marital status	Single vs. Married				.737	.591–.920	.007	.726	.575–.917	.007	.792	.642–.976	.029
Age	≤35 vs. > 35 years				.703	.569–.869	.001						

## Discussion

The present study is the first to investigate preventive behaviors throughout Iran. It was conducted when there were more than 27,000 patients and 2000 mortality cases due to COVID-19 according to the official statistics, given by the authorities. All Schools, Universities, and public places were closed for a month, before this study, and quarantine was imposed on all. We studied preventive behaviors and the personal and social factors affecting the target behaviors in a sample of candidates of over 15 years of age.

COVID-19 can spread from person to person. Many of the carriers of the virus show absolutely no symptoms and they are the main reason behind the spread of the virus. The virus can spread through droplets as well as direct contact with infected persons. Therefore, Personal Preventive Equipment (PPE), can contribute to controlling the spread [14, 15].

A study on the MERS Coronavirus proved that the people who are more prone to the infection showed better preventive behaviors. The results complied the theory that suggests the risk perception of infectious diseases promotes proper preventive behaviors. This suggests that risk perception can contribute to controlling the spread [16]. Socio-demographic status plays an important role in risk perception [17, 18].

Among the individual preventive behaviors, the most emphasized ones (Washing the hands for at least 20 s, wearing a mask, and wearing gloves) were studied separately. As it has been mentioned in the results, only 61.9% of the participants declared that they always wash their hands for at least 20 s. As hand-washing has always been important behavior related to personal health, there have been many studies in this field. Borchgrevink et al. conducted a study on 3749 individuals and concluded that only 5% of the people wash their hands properly and for more than 15 s [19]. These studies showed that insufficient attention is being paid to this preventive behavior.

A significant relation was realized to exist between the demographic status of gender, education and economic status, and the preventive behaviors of hand-washing, wearing masks, and protective gloves. This study showed that women have better preventive behavior comparison to men. Many studies conclude women are more cautious and preventive about infectious diseases. Moreover, housewives tend to have better preventive behavior [20–22]. Our results comply with a study by Wolf et al. which suggests that women have taken the new Corona Virus, more seriously, and people with lower economic status have not taken the disease seriously [23].

The results exhibited that younger people, men, single people, and those who belong to the lower levels of socio-economic status, have less preventive behaviors. These results comply with a Knowledge, Attitude, and Practice (KAP) study, conducted in China, during the outbreak [24] .It was reported that in research in Hong Kong, 61.2% of the participants, always wore masks to prevent SARS [20]. The use of PPE decreases the chances of the virus spreading; however, it is not definitive. With the rapid spread of the virus, PPE is harder to supply, and hence, the spread happens at an even faster pace [25]. On March 3, 2020, WHO requested all countries to increase their production of PPE by at least 40%, to meet the increased worldwide needs [26]. We believe that the reasons behind the lack of preventive behaviors in the mentioned groups could be due to lack of knowledge, lack of amenities, the belief of being resistant to the disease, and their higher potential for high-risk behaviors.

Social distancing and promotion of preventive behaviors are important solutions to break the chain of spread and flattening the disease curve. Educating the people about the concepts of the public health crisis promotes preventive behaviors among them, which in turn leads to the limitation of spread. Studies show that there is a chance of a repeated outbreak of the SARS virus; which can also be considered for the novel Corona Virus. Hence, it is necessary to educate and equip the public community with proper precautions to avoid a return of the epidemic. Moreover, Due to social distancing and limited physical contact, web-based data collection could be an advantage of the study.

## Limitations of the study

One of the limitations of this study, is the being self-reported, retrospective, and without external observation. Hence, there is a chance of bias, due to peer pressure. There is also a chance of recalling bias, due to the study being, retrospective. Furthermore, considering less access to internet connection and smartphones among the elderly and the group with lower economic status, this study might not be a perfect representation of the Iranian population. Hence, conducting a larger study would be a better representation for the elderly and those with lower economic status.

## Conclusions

The results show that according to the three main preventive behaviors of COVID-19 including hand-washing, wearing masks and gloves, 50 % of the population has not taken preventive behaviors seriously. It can also be concluded that these preventive behaviors have a significant relationship with some socio-demographic characteristics.

## Supplementary Information

**Additional file 1: Supplementary file**. Questionnaire of preventive behaviors during COVID-19 outbreak.

## Data Availability

Data is available on request from the corresponding author.

## Abbreviations

WHO: World Health Organization
PPE: Personal Preventive Equipment
