# Supplementary material for: COVID-19 preventive behaviors and influencing factors in the Iranian population; a web-based survey
Source: BMC Public Health. 2021 Jan 15;21:143. doi: 10.1186/s12889-021-10201-4 (PMC7809636; doi:10.1186/s12889-021-10201-4)
Supplement: Supplementary file 1 — Additional file 1: Supplementary file. Questionnaire of preventive behaviors during COVID-19 outbreak. [file 12889_2021_10201_MOESM1_ESM.docx]

How often do you consider each behavior during COVID-19 outbreak? Please put check mark (√)

| **Individual behaviors** | | | | | |
| --- | --- | --- | --- | --- | --- |
| I do NOT leave home, unless it is necessary | Always | Often | Sometimes | Rarely | Never |
| I avoid handshakes and hugging others |  |  |  |  |  |
| I keep a minimum distance of 1.5 meters from others |  |  |  |  |  |
| I avoid touching my face (eyes, nose and mouth) |  |  |  |  |  |
| I regularly wash my hands for AT LEAST 20 seconds |  |  |  |  |  |
| I wear disposable gloves when I leave the house |  |  |  |  |  |
| I cover my mouth and nose while sneezing or coughing |  |  |  |  |  |
| I dispose of tissue papers in a lidded trash can |  |  |  |  |  |
| I DO NOT visit friends and relatives |  |  |  |  |  |
| I DO NOT attend birthday parties, wedding parties, or any other parties |  |  |  |  |  |
| I DO NOT eat out |  |  |  |  |  |
| I DO NOT use public transportation |  |  |  |  |  |
| I disinfect my work space surfaces before anything else |  |  |  |  |  |
| **Practices when entering the house** | | | | | |
| I wash my hands before taking off my clothes or do any other task |  |  |  |  |  |
| I dry my hands using tissue papers |  |  |  |  |  |
| I dispose of the tissue paper in a lidded trash can |  |  |  |  |  |
| I disinfect my belongings such as cellphone, keys, wallet, etc. using alcohol disinfectant (70% alcohol) |  |  |  |  |  |
| I hang my clothes separately from other clothes when I enter the house |  |  |  |  |  |
| I wash my hands again, after removing my clothes |  |  |  |  |  |
| I wash my hands after using the WC and before eating |  |  |  |  |  |
| I disinfect all surfaces, everyday |  |  |  |  |  |
| **Practices when leaving the house** | | | | | |
| I ask myself about the necessity, when I am leaving the house |  |  |  |  |  |
| I take alcohol disinfectant with me |  |  |  |  |  |
| I wear disposable masks |  |  |  |  |  |
| I wear disposable gloves |  |  |  |  |  |
| I do not leave the house in case I have symptoms of fever and cough |  |  |  |  |  |
| I always carry clean tissue papers |  |  |  |  |  |
| I do not touch elevator buttons with bare hands |  |  |  |  |  |
| **Practices while using personal belongings** | | | | | |
| I do not take my cell phone out of my pocket when outside, unless there is an emergency |  |  |  |  |  |
| I do not remove my glasses or wrist-watch when outside |  |  |  |  |  |
| I do not place my belongings on surfaces I am not certain of their hygiene |  |  |  |  |  |
| I take food (Meat, chicken, eggs, etc.) only in a well done doneness |  |  |  |  |  |
